# Comparison of the Scorpionism Caused by *Centruroides margaritatus*, *Tityus pachyurus* and *Tityus* n. sp. aff. *metuendus* Scorpion Venoms in Colombia

**DOI:** 10.3390/toxins13110757

**Published:** 2021-10-25

**Authors:** Leydy Lorena Mendoza-Tobar, Ivonne Alejandra Meza-Cabrera, Juan C. Sepúlveda-Arias, Jimmy Alexander Guerrero-Vargas

**Affiliations:** 1 Programa de Doctorado en Ciencias Biomédicas, Facultad de Ciencias de la Salud, Universidad Tecnológica de Pereira, Pereira 660003, Colombia; l.mendoza@utp.edu.co (L.L.M.-T.); jcsepulv@utp.edu.co (J.C.S.-A.); 2 Grupo de Investigaciones Herpetológicas y Toxinológicas, Centro de Investigaciones Biomédicas-Bioterio, Departamento de Biología, Facultad de Ciencias Naturales, Exactas y de la Educación, Universidad del Cauca, Popayán 190002, Colombia; 3 Compañía de Patólogos del Cauca, Facultad de Ciencias de la Salud, Universidad del Cauca, Popayán 190002, Colombia; ivonneameza@gmail.com

**Keywords:** scorpionism, *Tityus pachyurus*, *Centruroides margaritatus*, *Tityus* n. sp. aff. *metuendus* histopathology, phyisiopathology, Colombia

## Abstract

Among other scorpion species, Colombia has two genera of the Buthidae family *Centruroides* and *Tityus,* considered to be dangerous to humans. This research shares scientific knowledge aiming to a better understanding about the pathophysiological effects of such venoms. The venom of the three species: *Centruroides margaritarus*, *Tityus pachyurus*, and *T*. n. sp. aff. *metuendus* with biomedical interest were studied. An initial pre-glycemic sample was taken from ICR mice. They were later intraperitoneally inoculated with doses of 35% and 70% of LD_50_ of total venom. Poisoning signs were observed during a 6-h period to determine the level of scorpionism. After observation, a second glycemic sample was taken, and a histopathological evaluation of different organs was performed. This work revealed that all three venoms showed considerably notorious histopathological alterations in main organs such as heart and lungs; and inducing multiple organ failure, in relation to the glycemia values, only *C. margaritatus* and *T.* n. sp. aff. *metuendus* showed significant changes through manifestation of hyperglycemia. According to the Colombian scorpionism level; signs were mild to severe affecting the autonomous nervous system.

## 1. Introduction

Scorpionism is defined as a disease caused after a scorpion sting to humans. It is considered a public health problem in tropical and subtropical regions [1,2,3,4] which is the reason why knowing geographical variability, scorpion species behaviour; and understand composition and biological activity of its venom, is very important to understand clinical complications observed in venoming accidents towards developing effective anti-venoms [5,6,7,8,9,10,11].

The most recent estimate of scorpion taxonomic diversity classifies 19 recognized families and 2437 species. There are about 80 species of scorpions in Colombia classified into four families, holding a particular medical interest, those belonging to the Buthidae family from the genus: *Centruroides* and *Tityus,* due to the frequency and severity of the scorpionism they cause [3,12,13,14,15,16,17].

Colombia includes four categories of scorpionism with some of the following symptoms: (A) Local manifestations: paresthesia, sweating; erythema, local pain; and hyperesthesia. (B) Mild symptoms: headache, nausea; pallor, sialorrhea; rhinorrhea, and odynophagia. (C) Moderate systemic manifestations: confusion, psychomotor agitation; ataxia, diarrhea; dystonia, priapism, and pancreatitis. (D) Severe systemic manifestations: ventricular arrhythmias, hypotension; bradycardia, cardiovascular collapse, pulmonary edema; neurological compromise (coma) and convulsive status [2,3,18,19].

In a sting accident, a scorpion injects its venom into the subcutaneous space. From there, it spreads into the central circulatory system, quickly transporting the toxins, to different tissues and organs such as kidneys, liver, lungs; muscles and heart [20].

Prognostic medical factors related to the severity of scorpion accidents are related to different aspects such as: (A) Toxicity: degree of toxicity of the injected venom. (B) Patient age: from severe to lethal on children under seven years of age; or elderly [21,22]. (C) Patient susceptibility to toxins [23]. (D) Species and specimen size: *Centruroides* and *Tityus* as the main genera related to more systemic effects, even death. In some cases, the size of the scorpion sting is related to a greater amount of injected venom; hence, the effect on the victim. (E) Finally, the time between the accident and access to hospital care, as it was found that, there is a close relationship between the symptoms manifestation and delay on obtaining medical care [24].

There are no regulations or epidemiological surveillance programs in Colombia for scorpion accidents that force healthcare centers to report scorpionism cases. In addition, few studies have been carried out in the country showing the prevalence of these cases of scorpionism, which reflects the fact that is an emerging health problem.

**Figure 1:**
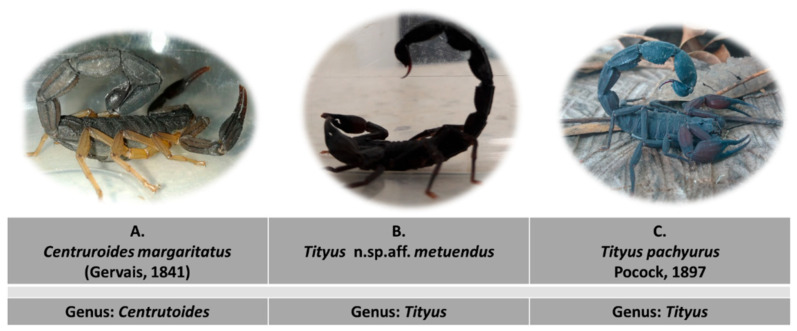
Scorpions of biomedical interest studied in this work, belonging to the genus *Centruroides* (**A**) and *Tityus* (**B**,**C**).

There is a need to characterize, at a clinical and medical level, the scorpionism caused by the venom of highly dangerous species: *Centruriodes margaritatus* (Figure 1) which is found in the southwestern regions of Colombia, specifically in Valle del Cauca; Cauca, and Nariño, where it is considered potentially dangerous to humans [3]; *Tityus pachyurus* (Figure 1) inhabits dry forests in the middle sector of the Magdalena River Valley (central Colombia), across the departments of Tolima, Cundinamarca, Boyacá, Antioquia, and Huila, this species has one of the most toxic venoms in Colombia and it causes the highest percentages of patient admissions in hospitals and health centers in the country [3,22,25]; and one of the most recent species found in Cauca state: *Tityus* n. sp. aff. *metuendus* (Figure 1) together with *T. pachyurus* it has one of the most toxic venoms and in some regions in Cauca State it presents a sympatric distribution along with *C. margaritatus* from which little information is known.

We seek to consolidate information about toxinology and biomedicine on this field aiming for a better understanding on suitable clinical handling of scorpionism towards optimization of treatments improving life quality on patients.

## 2. Results

### 2.1. Lethal Dose 50 (LD_50_)

The LD_50_ was obtained when mice were inoculated with total venom: 19.56 mg/kg of *C. margaritatus* venom; 2.92 mg/kg of *T. pachyurus* venom; and 1.012 mg/kg from *T.* n. sp. aff. *Metuendus* venom. Table 1 shows comparative LD_50_ of the total venom Vs the soluble phase (supernatant) reported [24,26,27].

### 2.2. Clinical In Vivo Effects of Envenomation

All Mice (five to each dose of scorpion venom) showed several intoxication symptoms after being inoculated with different dose (35% and 70% of the LD_50_) from the three studied scorpion species (Table 2).

Among the signs observed in *Centruroides margaritatus* envenoming, epiphora and death occurs under the highest dose of 70% LD_50_. Other signs were observed both at the 35% LD_50_ and 70% LD_50_. The first signs such as priapism, piloerection, hypoactivity, tachypnea, and sialorrhea were evident in the first half hour after inoculation with venom in both 35% LD_50_ and 70% LD_50_. Epiphora sign was evident during the first 30 min after inoculation with 70% dosage, while milky ocular discharge was observed around 90 min after venom inoculation regardless of dosage. About measuring pain, this intensified as time passed and was consistent with the observed lordosis. Surviving mice, however, evidenced improvement with respect to their expression of pain, approximately 5 h during observation. All signs persisted for 6 h. Regarding the two biomodels that died, one of them died 90 min after inoculation of the venom and the second one, 4 h after envenomation. Such event could be possible due to hypersensitivity to toxins.

According to the signs caused by Tityus pachyurus envenoming, epiphora and fast limb shiverings was observed only at the 70% LD_50_, all other signs were observed regardless of dose. Priapism, piloerection and hypoactivity, were evident on the first 30 min after venom inoculation independently from dose. Piloerection and hypoactivity persisted on average 5 h after inoculation of the 35% LD_50_ and until the end of the observation at the 70% LD_50_; while priapism disappeared on average three and a half hours after inoculation regardless of dosage. Tachypnea sign was evident from 60 min after inoculation with venom and persisted until the end of the observations. Sialorrhea appeared after 60 min with an average duration of one hour approximately independent of dose. Regarding the measurement of pain, this began to be evident 60 min after inoculation independent of the dose and was constant during 5 h observation with a later improvement. Lordosis was parallel to pain. In reference to the rapid tremor of the extremities, sign observed only in mice inoculated with 70% LD_50_, 90 min after the venom was inoculated. Diarrhea became evident between 2 and 3 h after envenoming independent of dose.

Envenoming with *Tityus*. n. sp. aff. *metuendus* showed fewer signs over biomodel compared to the other two species studyed. Signs such as piloerection, tachypnea, and seizures occurred independently of the dose; while hypoactivity, lack of grooming, and rapid tremors of extremities occurred only in mice inoculated with 70% LD_50_. As in the other two envenomings, piloerection appeared during the first 30 min after inoculation, and was persistent until the end of the observation. Tachypnea was a sign that, independently of dose, appeared during the first hour after the inoculation and had a mean duration of 3 to 4 h. Seizures and shiverings occurred sporadically on biomodels at different intervals, however, limbs shiverings were only evident at the 70% LD_50_.

### 2.3. Histophatological Effect

**Figure 2:**
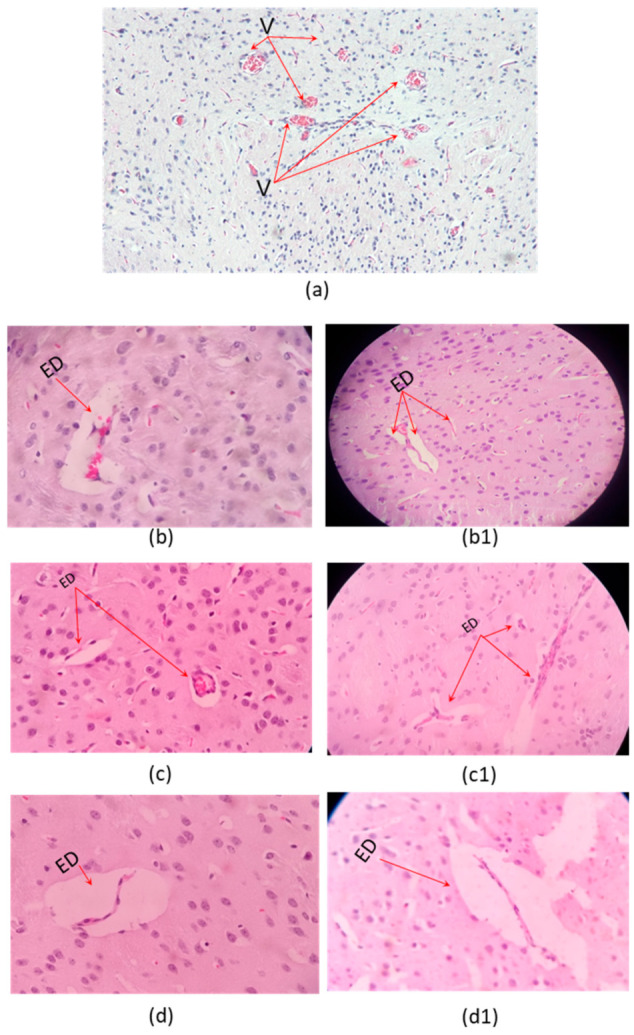
Histopathological changes on mice brain. (**a**) Control tissue (**b**–**d**), envenoming with 35% LD_50_; (**b1**,**c1**,**d1**) envenoming with 75% LD_50_ of *C. margaritatus*, *T. pachyurus*, and *T*. n. sp. aff. *metuendus* venom, respectively. ED: Edema. V: blood vessels. Control 10× magnification and the others one 40× magnification, hematoxylin-eosin stain.

**Figure 3:**
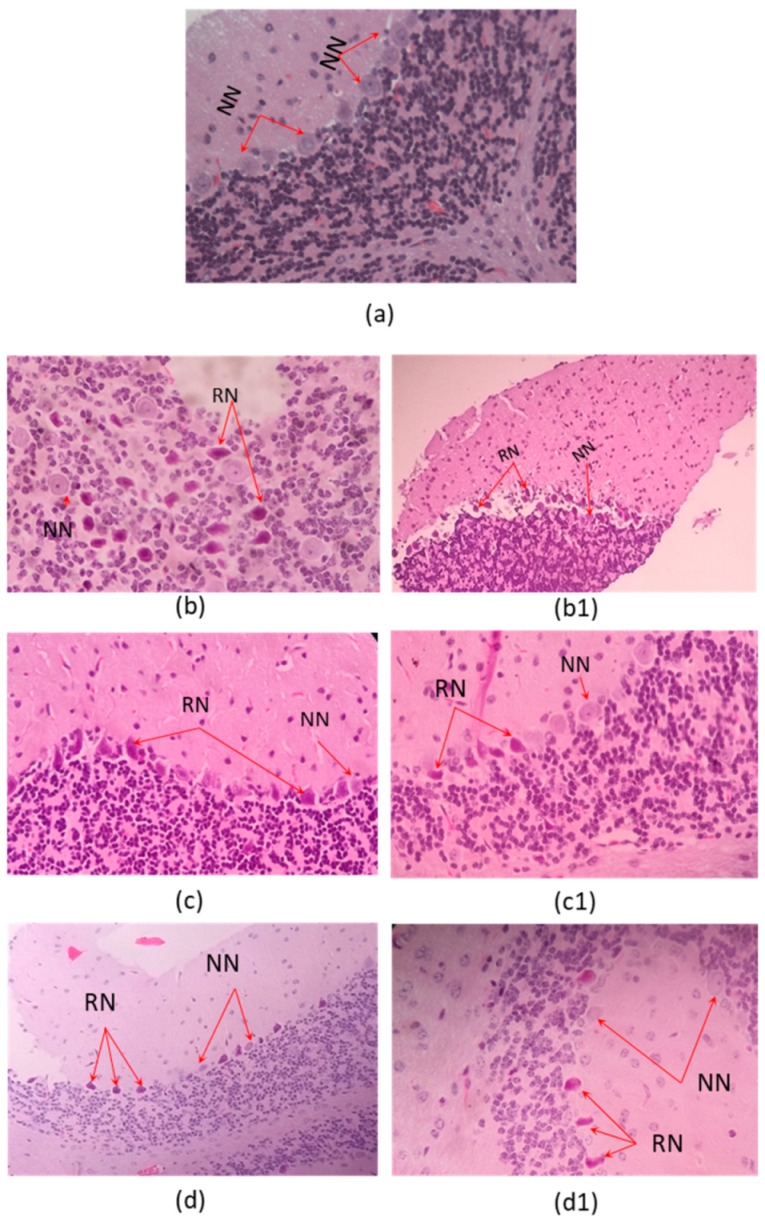
Histopathological changes on mice cerebellum. (**a**) Control tissue; (**b**–**d**). Envenoming with 35% LD_50_; (**b1**,**c1**,**d1**) envenoming with 75% LD_50_ of *C. margaritatus, T. pachyurus* and *T*. n. sp. aff. *metuendus* venom respectively. NN: normal neuron. RN: red neuron. (**b1**,**d**) are 10× magnification the other ones 40× magnification, hematoxylin-eosin stain.

**Figure 4:**
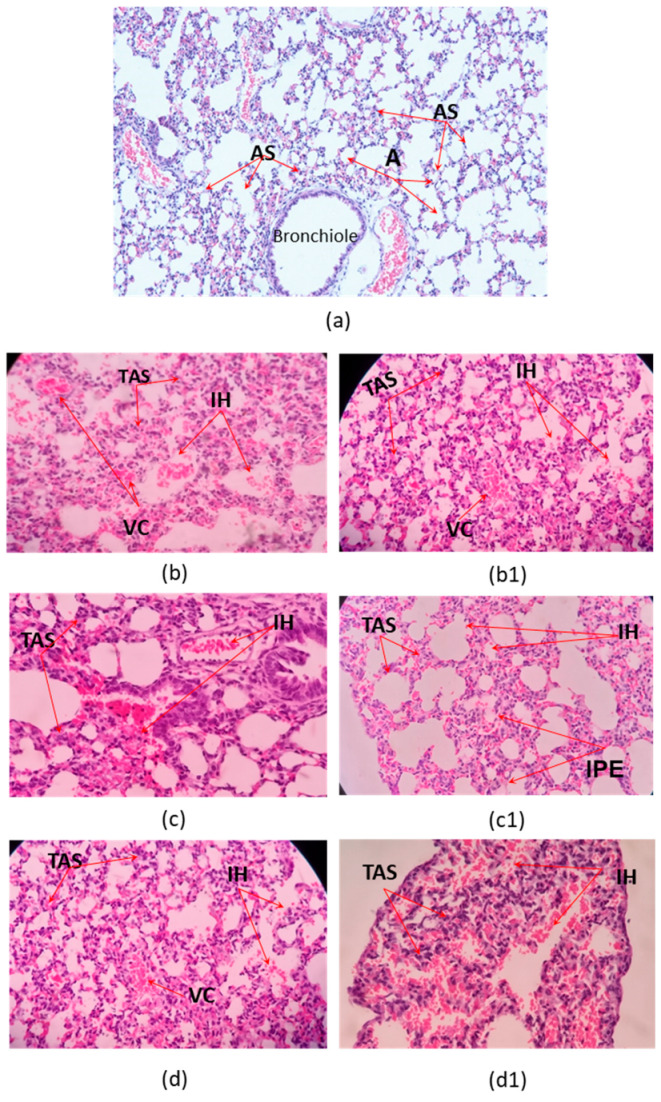
Histopathological changes on mice lungs. (**a**) Control tissue; (**b**–**d**). Envenoming with 35% LD_50_; (**b1**,**c1**,**d1**) envenoming with 75% LD_50_ of *C. margaritatus, T. pachyurus* and *T*. n. sp. aff. *metuendus* venom respectively. AS: Alveolar septa; A: Alveoli; VC: vascular congestion; TAS: thickening of the alveolar septa; IH: intra-alveolar hemorrhage; IPE: interstitial pulmonary edema; 40× magnification, hematoxylin-eosin stain.

**Figure 5:**
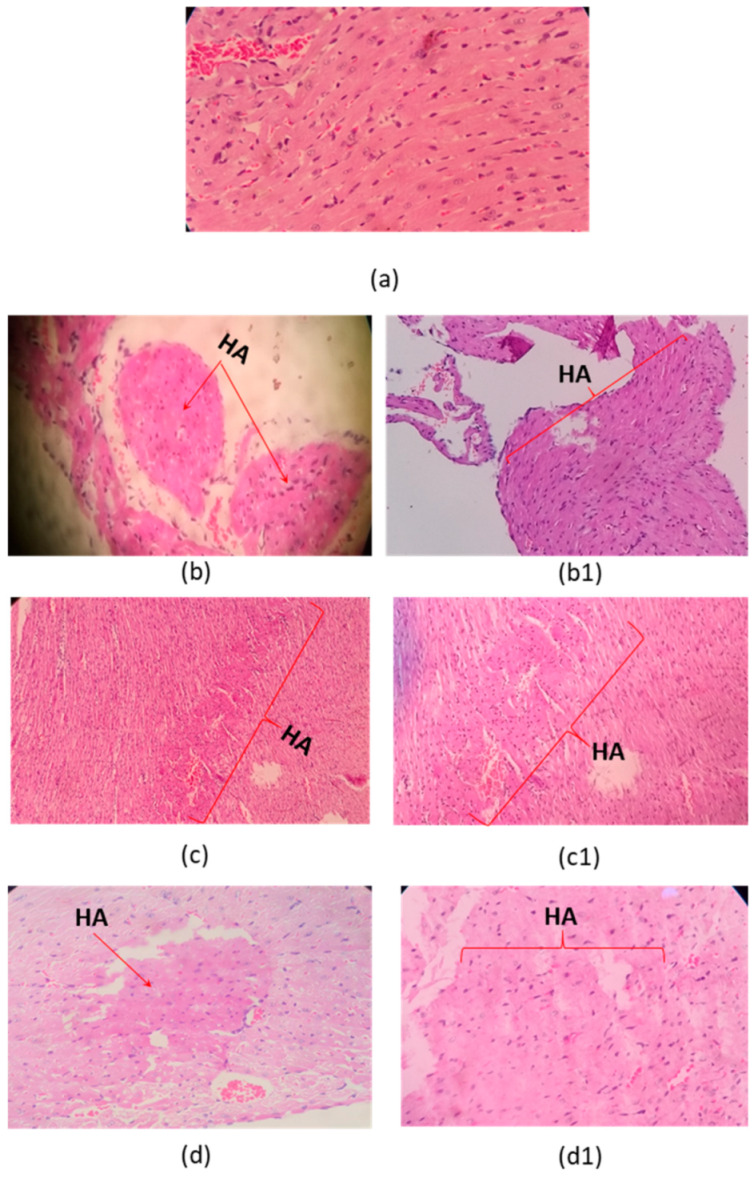
Histopathological changes on mice heart. (**a**) Control tissue; (**b**–**d**). Envenoming with 35% LD_50_; (**b1**,**c1**,**d1**) envenoming with 75% LD_50_ of *C. margaritatus* (Right ventricle); *T. pachyurus* (left ventricle), and *T*. n. sp. aff. *metuendus* (left ventricle) venom respectively. HA: heart attack outbreaks (**b1**,**c**,**c1**) under 10× magnification. Others with 40× magnification, hematoxylin-eosin stain.

**Figure 6:**
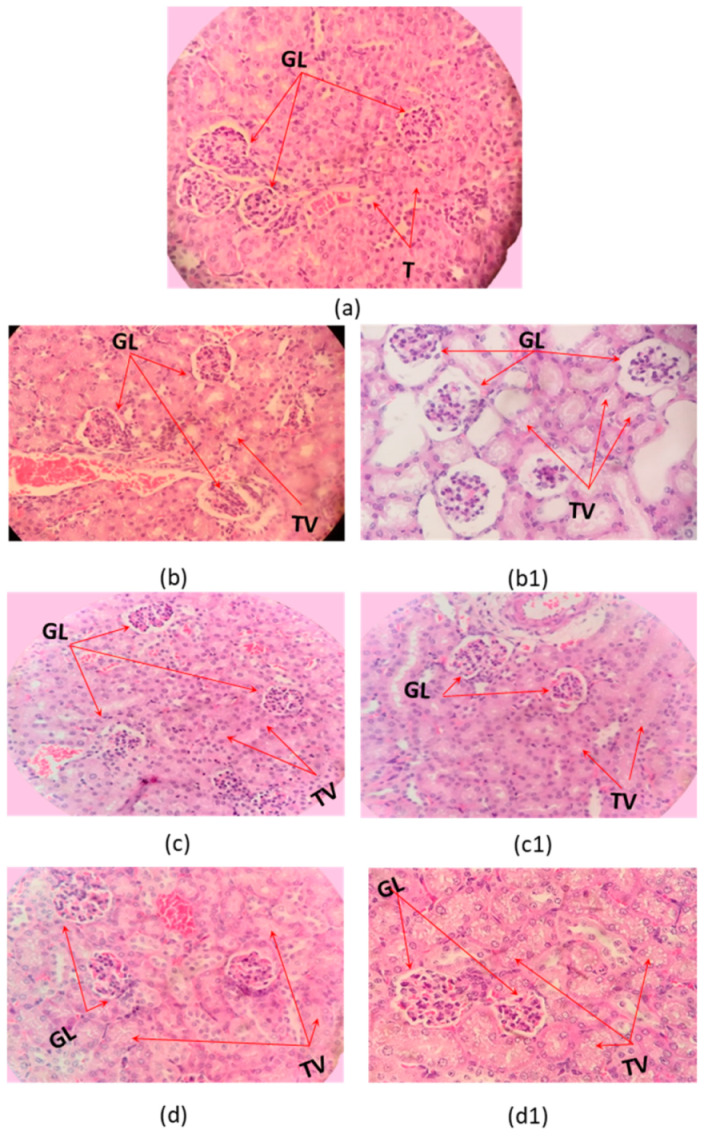
Histopathological changes on mice kidneys. (**a**) Control tissue; (**b**–**d**). Envenoming with 35% LD_50_; (**b1**,**c1**,**d1**) envenoming with 75% LD_50_. of *C. margaritatus, T. pachyurus* and *T*. n. sp. aff. *metuendus* venom respectively GL: glomerulus; TV: tubular vacuolization; 40× magnification, hematoxylin-eosin stain.

**Figure 7:**
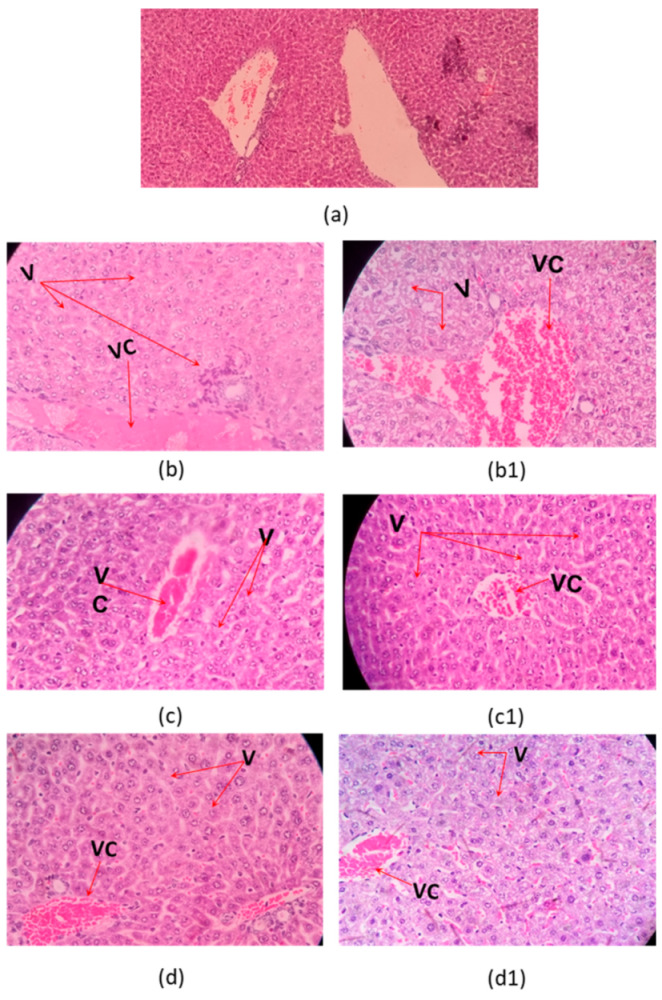
Histopathological changes on mice liver. (**a**) Control tissue; (**b**–**d**) envenoming with 35% LD_50_ (**b**–**d**) and 75% LD_50_ (**b1**,**c1**,**d1**) of *C. margaritatus*; *T. pachyurus*, and *T.* n. sp. aff. *metuendus* venoms, respectively. VC: vascular congestion; V: vacuolization; 10× control, the others one 40× magnification, hematoxylin-eosin stain.

Microscopic examination of organs after two sublethal dose (35% and 70% DL_50_) of the *C. margaritatus*; *T. pachyurus*; and *T*. n. sp. aff. *metuendus* venom, on a 6 h time period, showed evident changes in the brain (Figure 2), cerebellum (Figure 3); lung (Figure 4), heart (Figure 5); kidneys (Figure 6) and liver (Figure 7) compared with the control group mice.

Brain showed vascular congestion, edema and unusual presence of red neurons in the cerebellum (Figure 2). Hearth tissue showed infarct outbreaks and vascular congestion (Figure 5).

Lungs of three envenomed mice showed vascular congestion and thickening of the alveolar septa, and intra-alveolar hemorrhage (Figure 4). Might be related to cardiac alterations such as the presence of heart attack outbreaks (Figure 5).

Mice inoculated with the venom from *C. margaritatus* (Figure 6b,b1), and *T*. n. sp. aff. *metuendus* (Figure 6d,d1) exhibit kidney deterioration of Bowman’s capsules around the glomerulus and vacuolization of tubules. Additionally, is important to highlight that, this phenomenon is proportional to the applied dose (35%DL_50_ or 70% DL_50_). *T. pachyurus* (Figure 6c,c1) venom induced mild but evident degeneration in the Bowman’s capsules after exposure to the highest dose. Renal tubule vacuolization was also observed on all three studied venoms regardless of dose (Figure 6). Types of venom analyzed caused a slight increase in vascular congestion on liver, as well as the presence of cytoplasmic vacuoles exhibiting the development of ischemia (Figure 7). All histopathological signs occurred 6 h after intraperitoneal injection and regardless of dosage.

### 2.4. Glycemia Level

**Figure 8:**
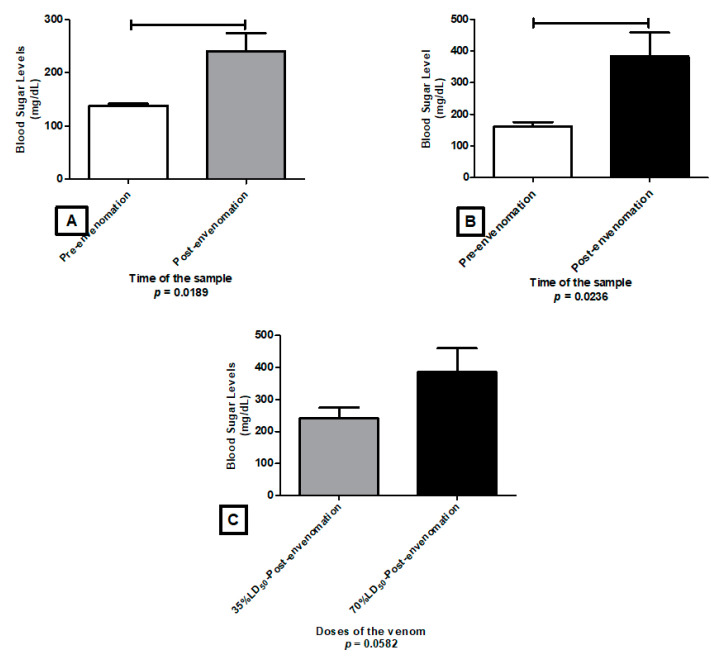
Glucose levels changes caused by the venom of *Centruroides margaritatus* in ICR mice, after envenomation with 35% LD_50_ (**A**) and 70% LD_50_ (**B**); and the comparison of the glycemic levels induced by both doses (**C**).

Post-envenoming with 35% LD_50_ and 70% LD_50_ of *C. margaritatus*, glycemia was significantly higher compared to the levels before exposure (Figure 8A,B). However, comparing glycemia levels post-exposure on both doses, no significant differences were found (Figure 8C) indicating that the increase of glucose levels is independent from the dose.

**Figure 9:**
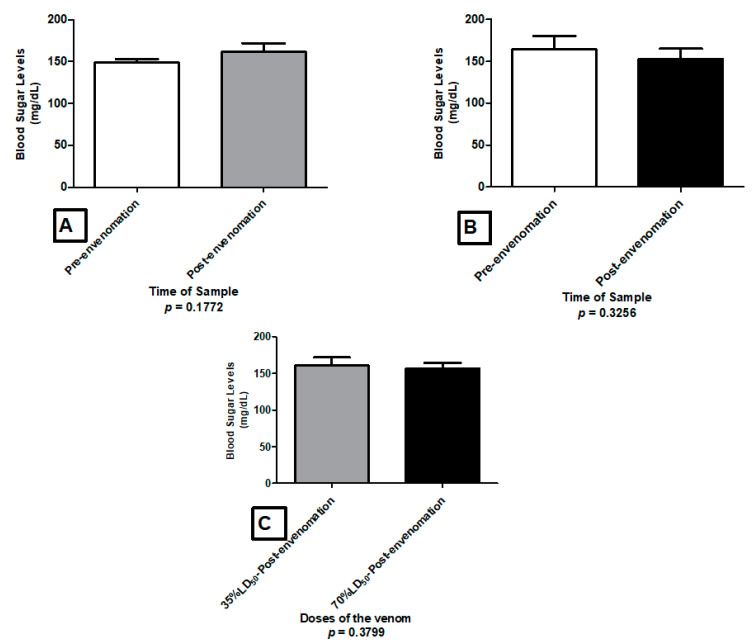
Alteration of glucose levels caused by the venom of *T. pachyurus* in ICR mice, after envenomation with 35% LD_50_ (**A**) and 70% LD_50_ (**B**), and the comparison of the glycemic levels induced by both dose (**C**).

None of *T. pachyurus* venom dosages induced alterations on blood glucose levels (Figure 9).

**Figure 10:**
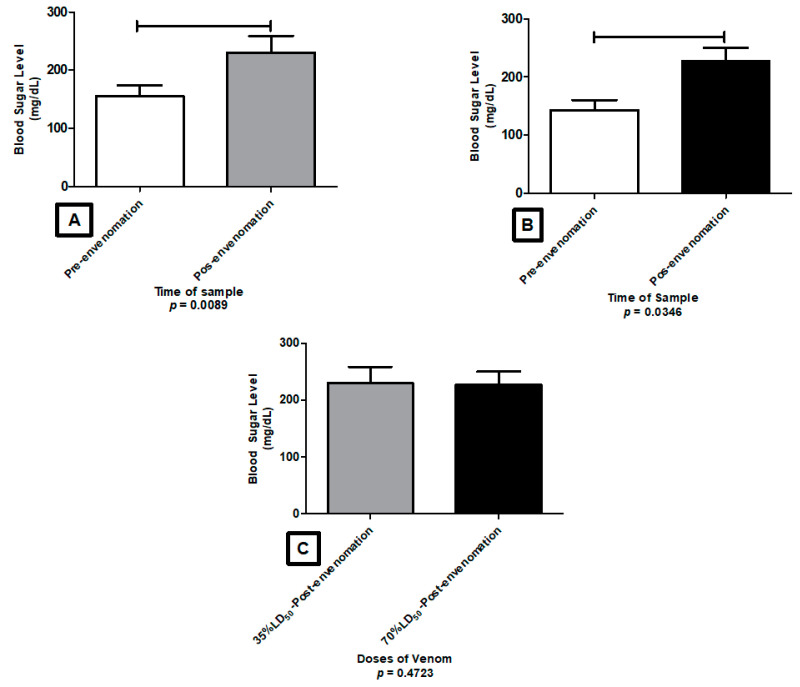
Alteration of Glucose levels caused by the venom of *T.* n. sp. aff. *metuendus* in ICR mice, after envenomation with a 35% LD_50_ (**A**) and 70% LD_50_ (**B**), and the comparison of the glycemic levels induced by both doses (**C**).

Regarding *T*. n. sp aff *mertuendus*, significant alterations were found between the pre and post dose of 35% LD_50_ (Figure 10A) as well as pre and post 70% LD_50_ (Figure 10B). However, there was not a statistical significance between 35% LD_50_ and 70% LD_50_ levels (Figure 10C), which indicates that the increment is not directly related to the concentration of the tested dose.

## 3. Discussion

It is relevant to consider that, working with the total venom—not only with the supernatant, but envenoming signs will also be closer to real conditions of a scorpion sting, therefore, experimentation will not exclude important components of its toxicity. LD_50_ differentiation might happened because, after going through centrifugation process, proteins or other constituents of high molecular weight could be loss, then affecting real results. It is also significant to acknowledge the variability of venom toxicity from an interspecific view, to explain the differential distress caused by each of the three species.

The scorpion poisoning signs observed with the three venoms evaluated on a murine model, allowed us to conclude that there is an alteration in the sympathetic and parasympathetic activity of the autonomic nervous system as described in Table 2. Additionally, symptoms described in mice revealed mild to severe envenoming, according to the approved Colombian scale of scorpionism [29].

Clinical symptoms of scorpion envenomation vary according to the species and the applied venom dose. Overall, signs such as pilomotor reflex, tachypnea, hypo-activity were present in all three species. However, symptoms such as epiphora and death were distinctively seen on 70% LD_50_ for *C. margaritatus. T. pachyurus* manifested a distinct symptom such as diarrhea; and *T.* n. sp. aff. *metuendus* envenoming showed seizures, however, the symptomatic profile on *T. pachyurus* and *C. margaritatus* was more alike between them, than among the species of the same genus, evidencing the complexity and variability of scorpion venom components.

Histopathologically, different samples of organ tissues were obtained to estimate relevant changes on altered organs. Only repetitive and common changes were considered for this analysis, excluding those associated with a single outcome.

Brain edema shown in Figure 2 is related to toxins exposure and the overall hypoxic-ischemic injury induced by the venom. Red neurons detected on the cerebellum (Figure 3) confirm this finding as they are sensitive to hypoxia and is associated with toxic lesions of the central nervous system. The morphological characteristics consist of a decrease in the size of the body cell, pyknosis of the nucleus, disappearance of the nucleolus, and loss of the substance of Nissl [30]. Such a condition is related to cardiac arrest and pulmonary disturbance (Figure 4 and Figure 5).

Independently from the dose, all three envenomings showed brain edema. However, it is important to highlight that these were minor lesions possibly related to time of exposure.

The histopathological changes in the heart (Figure 5) are due to hypoxia-ischemia. The typical lesions due to necrosis are recognized because wavy fibers can be found on the periphery of the infarction area. Such change could be due probably to the strong systolic pulling suffered by viable fibers located close to dead fibers with no contractile power. They look distended, curly with the intense eosinophilia of necrotic cells [31].

Pulmonary edema on scorpionism caused by *T. pachyurus* and *T. asthenes* has been previously reported [19,22,25,32]. This research found histopathological changes associated with pulmonary edema in the 70% LD_50_ of *Tityus pachyurus.* This condition could be explained because of a toxin lesion over the pulmonary micro-circulation. Edema happens mainly because an endotelium vascular lesion or due to the alveolar epithelial cells which produces a leak of liquids and proteins, into the intersticial space or into alveolly [33]. There were also other signs such as septum thickening, collapsed alveoli; and enlarged and congested vessels (Figure 4). These signs may be connected to tachypnea suggesting, the beginning of this pathology probably becoming visible if the exposure time increases.

Studies had shown that observations in brain, heart and lungs are linked to a sodium channel scorpio toxin (NaScTx) accumulation and activity possibly moving through the hemato-encephalic barrier, reaching the brain, and affecting the central nervous system, especially in young mice as in present study. The toxic effects of scorpion envenomation are due to a massive release of sympathetic and parasympathetic neurotransmiters such as catecholamines and acetylcholine, to which, according to the affected system -cholinergic or adrenergic, could produce arrythmias, cardiac failure or acute pulmonary edema [34,35,36,37]. This could explain the neurogenic hypothesis of the pulmonary edema in *Tityus pachyurus* as well as other alterations related to hypoxic-ischemic damage observed.

To build upon previous information on how scorpion venom causes such toxicity, venom from new world scorpions *Tityus serrulatus* and *Centruroides noxiu* causes a delay over the sodium channels gating on cardiac cells in neonate rats [38]. On the other hand, old world scorpion *Leiurus quinquestriatus hebraeus*, experimental results indicate that, NaScTx (Lqh II, Lqh III and LqhαIT) delays gating of cell cardiac sodium channels [39]. In addition, the *AmmTx3* toxin from *Androctonus mauretanicus,* inhibits gating of potassium Kv4.2 channels on cardiac cells [40].

It is important to emphasize that neither in Colombia nor in other places where *C. margaritatus* or *Tityus* n. sp. aff. *Metuendus* inhabits, cases of pulmonary edema caused by them were reported. However, some cases from Mexico region revealed severe and lethal cases for the *Centruroides* genus, predominant over *Tytius* for *C. elegans; C. infamatus; C. limpidus; C. tecomanus, C. noxius; C. pallidiceps, C. sculpturatus* and *C. suffusus* [41].

Concerning the cardiac tissue damage, Bolaños et al. (2013) [42], evaluated the cardiotoxic effect of *T*. n. sp. aff. *metuendus* venom on *Rattus norvegicus* rats. Individuals suffered signs of scorpion intoxication such as: paresthesia, epiphora; rhinorrhea, and piloerection also detected in this study.

Similarly, electrocardiographic records indicated the presence of a negative chronotropic effect with bradycardia. In addition, disturbances over the conductive system in hearth caused by the generation of atrial and ventricular fibrillation, and an extra-ventricular systole was detected. Atrial-ventricular blockage of first, second, and third degree was also observed, showing a robust cardiotoxic effect from this species venom.

Cardiac distress observed throughout the study caused by *T. pachyurus*, is consistent with a reported case from Tolemaida Cundinamarca. There, a 12 year old child showed strong skin local signs that quickly progressed into severe systemic symptoms such as myocardial dysfunction, cardiovascular collapse and cardiac arrest [43].

An electrocardiographic evaluation of *T. pachyurus* venom on *Rattus norvegicus,* evidenced the presence of bradycardia. It was followed by voltage changes of T wave raising without alterations on the conductive cardiac system. The plasma levels of the isoenzyme CK-MB significantly increased in a dose-dependent manner. The histopathological study showed interfibrillar edema, nodule myocarditis; hypertrophic myocytes, and noticeable vascular congestion. Such results indicate that *T. pachyurus* venom has cardiotoxic effects in experimental rats during the first 120 min after envenoming [44].

The vacuolization observed on kidneys is an ischemia sign connected to hypoxia caused by pulmonary and cardiac damage. This is due to a relative loss of oxygen because of the low blood supply, generating a mitochondrial deficiency which elevates the organelle’s reduced state because of an increment of the NADH:NAD+ ratio then, diminishing the ATP production from ADP. As a consequence, there is a reduction of the mitochondrial function as well as a fall at the intracellular enzymatic production inhibiting reactions related to water and electrolytes excretion causing enlargements seen as the observed vacuole [45].

This finding coheres with previous reports exhibiting a direct/indirect nephrotoxic outcome by the venom because, besides the information on last paragraph, it also produces the release of many modulators and mediators such as neurotransmitters, cytokines; oxygen-free radicals, and lipidic solutions [46,47,48]. This development is present in *C. margaritatus* [49], *Hottentota gentili* [50]; *T. nororientalis* [51], *Androctonus australis hector* [46]; *Androctonus liouvillei* [47], and *T. caripintensis* [48], along other specimens from the Buthidae family.

Animal studies had shown that scorpion venom is rapidly distributed from blood to tissues and is linked to a slow excretion because kidneys are the elimination path for such toxins. Kidneys presented the highest concentrations of toxins only 15 min after injection followed by liver, lungs, heart, and to a lesser degree on brain [52,53,54].

Concerning hepatic alterations, all three venoms induced a slight increment in vascular congestion. The presence of cytoplasmatic vacuoles also indicating steatosis, which consists of tiny lipid droplets in the cytoplasm of the hepatocyte (Figure 7). This might be due to hepatocytes damage as they become unable to phosphorylate the risen amounts of fatty acids, having as a consequence a fatty liver and alteration of the cell membranes of tissues. Scorpion venom possibly acts over the cell enzymatic mechanism involved in the metabolism of fatty acids, therefore, increasing their concentration towards the observed fatty liver. This result is coherent with previous studies using other venoms from species such as *Buthus lienhardi, Leiurus quinquestriatus and Buthus mionax*, where vacuolar damage was observed [55,56,57].

Regarding the glycemic levels, *C. margaritatus* and *T.* n. sp. aff. *mertuendus*, values were significantly different according to the control group (Figure 8). Pancreatic, thymus, and spleen alterations were not evident; however, it should be considered that such changes should take longer to appear over the functional changes reflected on glycemia.

*T. pachyurus,* did not induce alterations on glycemic levels on any dose. Pre- and post-values were not significantly different, against finds made by Barona et al. (2004). Probably because the inoculation they performed was a 0.5 LD_50_ subcutaneous inoculation.

Findings on this research agreed with an evidence of a 12 year old child who suffered a severe scorpionism by this species. Case also reported slight increase of glycemia of 131 mg/dL (Reference 70–110 mg/dL), without changes in pancreatic function [25].

The hyperglycemia observed during the first hours after envenomation is explained by the adrenergic reaction on the endocrine pancreas. Reports show that envenoming with *Tityus trivittatus*, such high glycemic levels persist during 24 h. This can be explained by a physiological alteration associated with a direct effect from toxins [58,59].

Pancreas is a combined exocrine and endocrine gland. On the one hand, its endocrine elements are the Langerhans islets (pancreatic islets) responsible for insulin production by β cells and glucagon by α cells; both, fundamental to carbohydrate metabolism and glucose homeostasis [60]. This study did not evaluate the direct cause of hyperglycemia induced by *C. margaritatus* or *T*. n. sp. aff. *metuendus*. However, some research had determined that the profuse liberation of catecholamines inhibits insulin secretion and the movement and breaking down of hepatic glycogen, generating hyperglycemia, helping to the myocardial damage [61,62,63,64].

Diverse works about the effects of scorpion venoms on humans as well as experimental animals, had determined that it induces a hyper/hypo-tension, tachycardia; hypothermia, leukocytosis; hyperglycemia, myocarditis; pancreatitis, trouble breathing; and other physiological alterations [20,22,65]. Such conditions resulting on the releasing of pro-inflammatory mediators might be connected to the cardiopulmonary alterations development and/or as sympathetic/parasympathetic stimuli on the autonomous nervous system because of the neurotoxins [20,66,67,68]—information that is consistent with some of our findings.

The action mechanisms on the scorpion venom toxicity differ depending on the venom components. However, the ones from the *Buthidae* family are considered with the highest toxicity for humans [69]. Among all scorpion venom components, toxins affecting the ionic channels are mainly responsible for humans’ envenoming [70], and are associated with changes observed during scorpionism, such as the presence of metalloproteins, involved with the development of pancreatitis [71,72,73]; or the existence of phospholipases PLA2, associated with the development of edema and erythrocytes hemolysis [74,75,76].

Colombian scorpions: *Centruroides margaritatus*, *Tityus pachyurus*, and *Tityus* n. sp. aff. metuendus have a venom that can cause severe scorpionism and strong histopathological damages as showed in this research. Previous works confirm this for, *Centruroides margaritatus* venom, also causing renal alterations [49], cardiovascular effects through alpha-1 adrenergic receptors [77]. It possesses a potent gamma potassium toxin with full-block activity on the hERG1 channel [78].

For *Tityus pachyurus* venom, the proteomic analysis pined out the potent toxin Tpa2 with biological activity on Na^+^ channels [79]. The transcriptomic analysis identify several toxins with biological action on Na^+^ channels [80] and show severe cardiovascular disfunction and pulmonary edema in humans [25]. This work reveals that *Tityus* n. sp. aff. *metuendus* venom had great toxicity under the LD_50_ causing severe scorpionism [3].

## 4. Conclusions

Throughout the research we can conclude that envenoming signs showed that these venoms can cause moderate to severe scorpionism because of systemic signs reflected on individuals, and histopathology test evidence.

Envenoming from all three species revealed hystopatological changes of great importance on brain, heart, lungs, liver, kidney during 6 h with the two sublethal dose. These findings reflect the development of a general ischemia caused mainly to the hypoxia event connected to the cardiac and pulmonary damage.

Related to the glycemia values, only *C. margaritatus* and *T.* n. sp. aff. *metuendus* showed significant changes through manifestation of hyperglycemia. This could be related to the changes on endocrine pancreas in charge of glucagon and insulin secretion playing a role on the glucose homeostasis in blood.

Pancreas, thymus and spleen alterations were not evident. However, it should be taken into account that, such changes take longer to develop according to the functional changes reflected on clinical testing of glycemia.

Symptomatology differences of the hystopatological damages and, blood sugar levels, confirm the evidence of inter-specific diversity of scorpion venoms highlighting the need to produce anti-venoms to neutralize scorpionism caused by Colombian species.

## 5. Materials and Methods

### 5.1. Mice

A total of 140 male ICR-CD1 mice (18–20 g) were obtained from the Barrera Bioterium from the National Health Institute to determine the LD_50_ and histopathological analysis, through intraperitoneal injection. The animals were kept at a constant room temperature and humidity, with a 12 h dark–light cycle. Food and water available ad libitum. The study was granted approval from the Ethics Committee of the Universidad del Cauca in record N° 6.1-1.25/010 (30 May 2018). All efforts were made to minimize the number animals tested and reduce their suffering.

### 5.2. Scorpions

Scorpion species were collected according to habitat from different regions in Colombia. A total 119 specimens belonging to *Centruroides margaritatus* were collected in Valle del Patía; and 30 specimens from *T.* n. sp. aff. *metuendus* were collected in Popayán, both locations in Department of Cauca. Further, 50 specimens were collected from *Tityus pachyurus* in Ibague, Department of Tolima.

They were kept at the Bioterio in Universidad del Cauca, under well ventilated wooden cages with food and water.

The *C. margaritatus* specie was determined according to identification key described by Bohórquez-Gómez [81]; *T. pachyurus*, by De Armas [12]; and *T.* n. sp. aff *metuendus* by Morales [27].

The venom was extracted by electrical stimulation, recovered in Eppendorf tubes, lyophilized and stored at −20 °C until use. For the experiments, two extractions were carried out with an interval of one month each; obtaining a total of 98 mg of venom from *C. margaritatus*, 62.2 mg of venom from *T. pachyurus* and 25.5 mg from *T.* n. sp. aff. *metuendus*

### 5.3. Determination of the Lethal Dose 50 (LD_50_)

Six serial dilutions for each venom of known concentrations were prepared. Five mice per dilution were inoculated intraperitoneally with 0.5 mL. Number of deaths was recorded for each group of individuals (dilution).

As an experimental requirement, both, 100% and 0% dilution are required. Reading of deaths, where 100% non-survival for all higher doses (minimum reading of 100% of deaths) and 0% where 0% deaths are presented for all lower doses (maximum reading of 0% of deaths). As a negative control, the same number of mice (5) were injected with saline solution for all tested individual survival.

Once the fulfillment of all the experimental requirements were verified, we proceeded to apply the statistical calculation tool of Spearman-Karber in order to estimate the value of the LD_50_ with its corresponding confidence interval (CI) [82].

This method of analysis is a standardized protocol, documented and supported by the INS (Integrated Management System). It is a routine procedure that performs the INS production direction under the name and code “determination of lethal dose fifty LD_50_”-MEN-R04.6020-011.

### 5.4. Histopathological Study

For histopathological study, seven groups of five (5) mice were intraperitoneal injected with two sublethal dose for each scorpion raw venom (with 35% LD_50_ and 70% LD_50_). Control group was injected with 0.9%saline solution.

During six hours period, signs were observed and registered. Pain was evaluated by means of the Grimace scale based on the facial expressions of the biomodels, where 0 indicates absence of pain; 1 moderately present pain, and 2 obviously present pain [83]. After six hours, mice were anesthetized with pentobarbital. Vital organs such as brain, lungs, liver, heart, kidneys, pancreas and spleen were dissected and fixed in 10% formalin solution. Organs were dehydrated in a grade alcohol series and embedded in paraffin wax. Sections of 10 μm thickness were stained with hematoxylin–eosin (HE) for pathological studies as described by Kiernan, 1999 [84].

### 5.5. Glycemia Level

Glycemia levels were taken before venom injection and 6 h after inoculation. A pre-venom value was determined as a control level for each individual. Levels were measured with an electronic glucometer OKmeter-MatchII. Sample measurements were carried out as described by manufacturer’s instructions.

## Figures and Tables

**Table 1:** Comparison of the total venom toxicity with the soluble phase venom (supernatant after centrifugation) toxicity of the *C. margaritatus*, *T*. n. sp. aff *metuendus*, and *T. pachyurus* scorpions.

Scorpion Specie	LD_50_ Total Venom (mg/kg)	LD_50_ Soluble Venom (mg/kg)	Administration
*C. margaritatus*	19.56	42.8 [26]	Intraperitoneal
*T. pachyurus*	2.92	4.8 [24]	Intraperitoneal
*T.* n. sp. aff *metuendus*	1.02	3.5 [28]	Intraperitoneal

**Table 2:** Comparison of envenomig signs with total venom toxicity of the *C. margaritatus*, *T. pachyurus* and *T.* n. sp. aff. *metuendus* Scorpions.Grimace Scale: 0 absence of pain; 1. Moderately present pain; and 2 obviously present pain.

*Centruroides margaritatus*	*Tityus pachyurus*	T. n. sp. aff. *metuendus*
Piloerection	Piloerection	Piloerection
Priapism	Priapism	
Hypoactivity	Hypoactivity	Hypoactivity (70% LD_50_)
Tachypnea	Tachypnea	Tachypnea
Sialorrhea	Sialorrhea	
Epiphora (70% LD_50_)	Epiphora (70% LD_50_)	
Grimace–pain score: 2 Lordosis	Grimace -pain score: 2 Lordosis	Grimace pain score: 1
Milky ocular secretion	Milky ocular secretion	
No Grooming	No Grooming	No Grooming (70% LD_50_)
Death (2–70% LD_50_)		
		Seizures
	Fast limb shiverings (70% LD_50_)	Fast limb shiverings (70% LD_50_)
	Diarrhea	

## Data Availability

All data are reported in the manuscript.

## References

[1] Chippaux J.-P.P., Goyffon M. (2008). Epidemiology of scorpionism: A global appraisal. Acta Trop..

[2] Khattabi A., Soulaymani-Bencheikh R., Achour S., Salmi L.-R.R. (2011). Classification of clinical consequences of scorpion stings: Consensus development. Trans. R. Soc. Trop. Med. Hyg..

[3] Guerrero-Vargas J.A., Rodriguez-Buitrago J., Ayerbe S., Florez-Daza E., Beltran-Vidal J.T., Gopalakrishnakone P., Possani L.D., Schwartz E.F., Rodriguez de la Vega R.C. (2015). Scorpionism and Dangerous Species of Colombia. Scorpion Venoms.

[4] Rodríguez-Vargas A. (2015). Casuística de Accidentes por Arácnidos Línea Nacional de Toxicología.

[5] Borges A., De Sousa L., Espinoza J., Santos R.G., Kalapothakis E., Valadares D., Chávez-Olórtegui C. (2008). Characterization of Tityus scorpion venoms using synaptosome binding assays and reactivity towards Venezuelan and Brazilian antivenoms. Toxicon.

[6] Borges A., Tsushima R.G., Backx P.H. (1999). Antibodies against Tityus discrepans venom do not abolish the effect of Tityus serrulatus venom on the rat sodium and potassium channels. Toxicon.

[7] Carmo A.O., Chatzaki M., Horta C.C.R., Magalhães B.F., Oliveira-Mendes B.B.R., Chávez-Olórtegui C., Kalapothakis E. (2015). Evolution of alternative methodologies of scorpion antivenoms production. Toxicon.

[8] Estrada-Gómez S., Gomez-Rave L., Vargas-Muñoz L.J., van der Meijden A. (2017). Characterizing the biological and biochemical profile of six different scorpion venoms from the Buthidae and Scorpionidae family. Toxicon.

[9] Estrada-Gómez S., Vargas-Muñoz L.J., Saldarriaga-Córdoba M., Quintana Castillo J.C. (2016). Venom from Opisthacanthus elatus scorpion of Colombia, could be more hemolytic and less neurotoxic than thought. Acta Trop..

[10] Pessini A.C., Takao T.T., Cavalheiro E.C., Vichnewski W., Sampaio S.V., Giglio J.R., Arantes E.C. (2001). A hyaluronidase from Tityus serrulatus scorpion venom: Isolation, characterization and inhibition by flavonoids. Toxicon.

[11] Horta C.C.R., de Magalhães B.F., Oliveira-Mendes B.B.R., Carmo A.O., Duarte C.G., Felicori L.F., Machado-de-Ávila R.A., Chávez-Olórtegui C., Kalapothakis E. (2014). Molecular, Immunological, and Biological Characterization of Tityus serrulatus Venom Hyaluronidase: New Insights into Its Role in Envenomation. PLoS Negl. Trop. Dis..

[12] De Armas L.F., Sarmiento D.L., Flórez-Daza E., Luna Sarmiento D. (2012). Composición del género Centruroides Marx, 1980 (Scorpiones: Buthidae) en Colombia, con la descripción de una nueva especie. Boletín Soc. Entomológica Aragon..

[13] Perafán C., Sabogal-González A., Moreno-González J.A., García-Rincón A., Luna-Sarmiento D., Romero-Ortíz C., Flórez-Daza E. (2013). Diagnóstico del estado actual de la fauna de arácnidos y de su gestión en Colombia. Memorias 40 Congr. Colomb. Entomol. SOCOLEN.

[14] Flórez-Daza E. (2001). Escorpiones de la Familia Buthidae (Chelicerata: Scorpiones) de Colombia. Biota Colomb..

[15] Florez E., Sanchez H., Rangel O. (1995). La Diversidad de Aracnidos en Colombia. Aproximación inicial. Colombia Diversidad Biótica I.

[16] Rein J.O. The Scorpion Files. http://www.ntnu.no/ub/scorpion-files/index.php.

[17] Lourenço W.R. (2018). The evolution and distribution of noxious species of scorpions (Arachnida: Scorpiones). J. Venom. Anim. Toxins Incl. Trop. Dis..

[18] Ayerbe S.G., Buitrago J.R., Correa L.F., Berdejo J.P., Mora V.H., Sánchez Alarcón D.M., Gutiérrez De Salazar M. (2008). Accidente Aracnidico. Guias Para el Manejo de Urgencias Toxicológicas.

[19] Gómez J.P., Quintana J.C., Arbeláez P., Fernández J., Silva J.F., Barona J., Gutiérrez J.C., Díaz A., Otero R. (2010). Picaduras por escorpión Tityus asthenes en Mutatá, Colombia: Aspectos epidemiológicos, clínicos y toxinológicos. Biomédica.

[20] D’Suze G., Salazar V., Díaz P., Sevcik C., Azpurua H., Bracho N. (2004). Histophatological changes and inflammatory response induced by Tityus discrepans scorpion venom in rams. Toxicon.

[21] Pineda D., Flórez E. (2002). Picaduras de escorpión. Accidente por Animales Venenoso.

[22] Otero R., Navio E., Céspedes F.A., Núñez M.J., Lozano L., Moscoso E.R., Matallana C., Arsuza N.B., García J., Fernández D. (2004). Scorpion envenoming in two regions of Colombia: Clinical, epidemiological and therapeutic aspects. Trans. R. Soc. Trop. Med. Hyg..

[23] Cesaretli Y., Ozkan O. (2010). Scorpion stings in Turkey: Epidemiological and Clinical aspects between the years 1995 and 2004. Rev. Inst. Med. Trop..

[24] Barona J., Otero R., Núñez V. (2004). Aspectos toxinológicos e inmunoquímicos del veneno del escorpión Tityus pachyurus Pocock de Colombia: Capacidad neutralizante de antivenenos producidos en Latinoamérica. Biomédica.

[25] Izquierdo L.M., Rodríguez Buitrago J.R. (2012). Cardiovascular dysfunction and pulmonary edema secondary to severe envenoming by Tityus pachyurus sting. Case report. Toxicon.

[26] Guerrero-Vargas J.A., Ayarbe S., Rada-Mendoza M., Vélez P., D’Suze G. Estandarización de la extracción de veneno del escorpión Centruroides margaritatus (Scorpionida: Buthidae), del municipio del Patía, determinación de la DL-50. Proceedings of the XXX Congreso Nacional de la Sociedad de Entomología.

[27] Morales-Duque H. (2011). Determinación de la Actividad Neurotóxica y Antimicrobiana del Veneno del Escorpión Tityus sp. (Buthidæ).

[28] Morales Duque H. (2013). Caracterização de Novos Peptidos Bloqueadores de Cananis Para K+ Isolados da Peçonha do Escopião *Tityus* sp..

[29] Ayerbe S., Rodríguez J.R., Correa L.F., Berdejo J.P., Mora V.H., Sánchez D.M., Gutiérrez M. (2008). Accidente Escorpiónico. Guias para el Manejo de Urgencias Toxinológicas.

[30] De Girolami U., Anthony D., Frosch M., Cotran R., Kumar V., Robins S. (2000). El Sitema Nervioso Central. Patologia Estructural y funcional.

[31] Schoen F., Cotran R., Kumar V., Robins S. (2000). El Corazón. Patologia Estructural y Funcional.

[32] Valderrama R. (1998). Envenenamiento por picadura de escorpiones. Prim. Simp. Colomb. Toxinología toxinas y envenenamiento por Anim. plantas Microorg..

[33] Konzik L., Cotran R., Kumar V., Robins S. (2000). Pulmón. Pastología Estructural y Funcional.

[34] Clot-Faybesse O., Guieu R., Rochat H., Devaux C. (1999). Toxicity during early development of the mouse nervous system of a scorpion neurotoxin active on sodium channels. Life Sci..

[35] Nunan E.A., Moraes M.F.D., Cardoso V.N., Moraes-Santos T. (2003). Effect of age on body distribution of Tityustoxin from Tityus serrulatus scorpion venom in rats. Life Sci..

[36] Nencioni A.L.A., Lourenço G.A., Lebrun I., Florio J.C., Dorce V.A.C. (2009). Central effects of Tityus serrulatus and Tityus bahiensis scorpion venoms after intraperitoneal injection in rats. Neurosci. Lett..

[37] Cupo P., Azevedo-Marques M.M., Hering S.E., Cardoso J.L.C., França F.O.S., Wen F.H., Málaque C.M.S., Haddad V. (2003). Escorpionismo. Animais Peçonhentos No Brasil. Biologia, Clínica e Terapêutica dos Acidentes.

[38] Yatani A., Kirsch G.E., Possani L.D., Brown A.M. (1988). Effects of New World scorpion toxins on single-channel and whole cell cardiac sodium currents. Am. J. Physiol. Hear. Circ. Physiol..

[39] Chen H., Heinemann S.H. (2001). Interaction of scorpion α-toxins with cardiac sodium channels: Binding properties and enhancement of slow inactivation. J. Gen. Physiol..

[40] Nicolas S., Zoukimian C., Meudal H., De Waard S., Ait Ouares K., Canepari M., Beroud R., Landon C., De Waard M., Boturyn D. (2019). In vitro and in vivo characterization of asynthetic scorpion toxin AmmTx3, a potentinhibitor of cardiac voltage-gated potassiumchannel Kv4.2. Arch. Cardiovasc. Dis. Suppl..

[41] Dehesa-Dávila M., Possani L.D. (1994). Scorpionism and serotherapy in Mexico. Toxicon.

[42] Bolaños C., Beltran J.T., Guerrero-Vargas J.A. Cardiotoxic evaluation of the *Tityus* sp. Scorpion venom on Rattus norvegicus. (Wistar rats). Proceedings of the XI Congress of the Pan-American Section of the International Society on Toxinology.

[43] Otero R., Uribe F., Sierra A. (1998). Envenenamiento escorpiónico en niños. Actual. Pediátr.

[44] Beltran J.T., Ayerbe A., Arenas C., Segura B., Torres M., Hurtado F., Morales H., Tobar Y., Coral R. Evaluación Cardiotóxica del Veneno del escorpion Tityus pachyurus (Pocock, 1897), en ratas Rattus norvegicus. Proceedings of the VIII simposio de Investigaciones en Ciencias Biológicas.

[45] De Oliveira N.A., Cardoso S.C., Barbosa D.A., da Fonseca C.D. (2021). Acute kidney injury caused by venomous animals: In-flammatory mechanisms. J. Venom. Anim. Toxins Incl. Trop. Dis..

[46] Saidani C., Béchohra L., Laraba-Djebari F., Hammoudi-Triki D. (2019). Kidney inflammation and tissue injury induced by scorpion venom: Comparison with a nephrotoxic model. Toxin Rev..

[47] El Hidan M.A., Touloun O., El Hiba O., Boumezzough A. (2016). Pathophysiological and neurobehavioral injuries in mice experimentally envenomed with Androctonus liouvillei (Pallary, 1928) scorpion venom. Exp. Toxicol. Pathol..

[48] Portillo A., Sojo I., Zerpa J. (1996). Alteraciones Histopatológicas Causadas por el Veneno del Escorpión Tityus Caripitensis (Familia: Buthidae) sobre Hígado y riñón de Ratón.

[49] Galíndez-Cerón J.D., Jorge R.J.B., Chavez-Acosta M.H., Jorge A.R.C., Alves N.T.Q., Prata M.M.G., Rodrigues F.A., Havt A., Sampaio T.L., Martins A.M.C. (2019). Renal Alterations Induced by the Venom of Colombian Scorpion Centruroides Margaritatus. Curr. Top. Med. Chem..

[50] El Hidan M.A., Touloun O., El Hiba O., Chait A., Eddine Hafid J., Boumezzough A. (2015). Behavioral, histopathological and biochemical impairments observed in mice envenomed by the scorpion: Hottentota gentili (Pallary, 1924). Toxicon.

[51] Salomón L. (2009). Cambios Histopatológicos Agudos Causados por el Veneno de Tityus Nororientalis (Scorpiones, Buthidae) en Riñones de Ratones NMRI, BALBc y C57BL/6..

[52] Alves R.D.S., Falcão Do Nascimento N.R., Ferreira Barbosa P.S., Kerntopf M.R., Abreu Lessa L.M., De Sousa C.M., Martins R.D., Sousa D.F., Rodrigues De Queiroz M.G., Toyama M.H. (2005). Renal effects and vascular reactivity induced by Tityus serrulatus venom. Toxicon.

[53] Ismail M., Abd-Elsalam M.A. (1988). Are the toxicological effects of scorpion envenomation related to tissue venom concentration?. Toxicon.

[54] Murugesan S., Radha Krishna Murthy K., Noronha O.P.D., Samuel A.M. (1999). Tc 99m—Scorpion Venom: Labelling, Biodistribution and Scintiimaging. J. Venom. Anim. Toxins.

[55] Ait Laaradia M., El Hidan M.A., Marhoume F., Bouimeja B., Oufquir S., Sokar Z., Boumezzough A., Chait A. (2018). Buthus lienhardi venom and pathophysiological effects at the histological, hematological, biochemical and motor skills levels. Toxicon.

[56] El-Asmar M.F., Soliman S.F., Ismail M., Osman O.H. (1974). Glycemic effect of venom from the scorpion Buthus minax (L. Koch). Toxicon.

[57] Muhammad S. (2011). The acute effects of scorpion (Leiurus quinquestriatus) venom on some clinicalpathological parameters in Guinea pigs Muhammad. J. Am. Sci..

[58] Ghersy de Nieto M.T., D’Suze G., Sevcik C., Salazar V., Silva V., Urbina H., Pardo R. (2004). Diabetes inducida por emponzoñamiento escorpiónico grave en un lactante de un año. Medicrit.

[59] Gordillo M. (2000). Escorpionismo en Pediatría. Arch. Argent. Pediatr.

[60] Bacon F., Greneser F. (2001). Apararto Digestivo. Histología.

[61] Babloul M., Kallel H., Rekik N., Hamida C., Chelly H., Bouaziz M. (2005). Atteinte cardiovasculaire lors d’envenimation scorpionique grave: Mécanismes et physiopathologie. Press. Med..

[62] De Roodt A.R. (2015). Veneno de escorpiones (alacranes) y envenenamiento. Acta Bioquímica Clínica Lat..

[63] Murthy K.R.K., Rao R.P., Natu V.S., Kumar Z.N., Lavanya M. (2015). Suppressed Insulin Secretion, Elevated Mediators of Inflammation, Hyper-Insulinemia-Insulin Resistance: Insulin Administration Reverses Cardiovascular, Metabolic Changes, Pulmonary Edema and All Other Clinical Manifestations in Scorpion Envenoming Syndrom. Indian J. Mednodent Allied Sci..

[64] Murthy K.R.K., Hase N.K. (1994). Scorpion envenoming and the role of insulin. Toxicon.

[65] D’Suze G., Moncada S., González C., Sevcik C., Aguilar V., Alagón A. (2003). Relationship between plasmatic levels of various cytokines, tumour necrosis factor, enzymes, glucose and venom concentration following Tityus scorpion sting. Toxicon.

[66] Mazzei C.A., Dàvila D.F., Donis J.H., Arata G., Villarreal V., Barboza J.S., Mazzei de Dàvila C.A., Dàvila D.F., Donis J.H., De Bellabarba G.A. (2002). Sympathetic nervous system activation, antivenin administration and cardiovascular manifestations of scorpion envenomation. Toxicon.

[67] Sofer S., Gueron M., White R.M., Lifshitz M., Apte R.N. (1996). Interleukin-6 release following scorpion sting in children. Toxicon.

[68] Ismail M. (1995). The scorpion envenoming syndrome. Toxicon.

[69] De la Vega R.C., Possani L.D. (2005). Overview of scorpion toxins specific for Na+ channels and related peptides: Biodiversity, structure–function relationships and evolution. Toxicon.

[70] Quintero-Hernández V., Jiménez-Vargas J.M., Gurrola G.B., Valdivia H.H., Possani L.D. (2013). Scorpion venom components that affect ion-channels function. Toxicon.

[71] Carmo A.O., Oliveira-Mendes B.B.R.R., Horta C.C.R.R., Magalhães B.F., Dantas A.E., Chaves L.M., Chavez-Olortegui C., Kalapothakis E., Chávez-Olórtegui C., Kalapothakis E. (2014). Molecular and functional characterization of metalloserrulases, new metalloproteases from the Tityus serrulatus venom gland. Toxicon.

[72] Ortiz E., Rendón-Anaya M., Rego S.C., Schwartz E.F., Possani L.D. (2014). Antarease-like Zn-metalloproteases are ubiquitous in the venom of different scorpion genera. Biochim. Biophys. Acta (BBA)-General Subj..

[73] Fletcher P.L., Fletcher M.D., Weninger K., Anderson T.E., Martin B.M. (2010). Vesicle-associated membrane protein (VAMP) cleavage by a new metalloprotease from the Brazilian scorpion Tityus serrulatus. J. Biol. Chem..

[74] Zamudio F.Z., Conde R., Arévalo C., Becerril B., Martin B.M., Valdivia H.H., Possani L.D. (1997). The mechanism of inhibition of ryanodine receptor channels by imperatoxin I, a heterodimeric protein from the scorpion Pandinus imperator. J. Biol. Chem..

[75] Conde R., Zamudio F.Z., Becerril B., Possani L.D. (1999). Phospholipin, a novel heterodimeric phospholipase A2 from Pandinus imperator scorpion venom. FEBS Lett..

[76] Valdez-Cruz N.A., Batista C.V.F., Possani L.D. (2004). Phaiodactylipin, a glycosylated heterodimeric phospholipase A2 from the venom of the scorpion Anuroctonus phaiodactylus. Eur. J. Biochem..

[77] Romero-Imbachi M.R., Cupitra N., Ángel K., González B., Estrada O., Calderón J.C., Guerrero-Vargas J., Beltrán J., Narvaez-Sanchez R. (2021). Centruroides margaritatus scorpion complete venom exerts cardiovascular effects through alpha-1 adrenergic receptors. Comp. Biochem. Physiol. Part C Toxicol. Pharmacol..

[78] Beltrán-Vidal J., Carcamo-Noriega E., Pastor N., Zamudio-Zuñiga F., Guerrero-Vargas J.A., Castaño S., Possani L.D., Restano-Cassulini R. (2021). Colombian Scorpion Centruroides margaritatus: Purification and Characterization of a Gamma Potassium Toxin with Full-Block Activity on the hERG1 Channel. Toxins.

[79] Barona J., Batista C.V.F., Zamudio F.Z., Gomez-Lagunas F., Wanke E., Otero R., Possani L.D. (2006). Proteomic analysis of the venom and characterization of toxins specific for Na+-and K+-channels from the Colombian scorpion Tityus pachyurus. Biochim. Biophys. Acta (BBA)-Proteins Proteomics.

[80] Guerrero-Vargas J.A., Mourão C.B., Quintero-Hernández V., Possani L.D., Schwartz E.F. (2012). Identification and phylogenetic analysis of Tityus pachyurus and Tityus obscurus novel putative Na-channel scorpion toxins. PLoS ONE.

[81] Bohórquez-Gómez R.M. (2016). Revisión Taxonómica y Distribución Geográfica de las especies de Tityus, Subgénero Atreus, (Scorpiones, Buthidae) Presentes en Colombia.

[82] Hamilton M.A., Russo R.C., Thurston R.V. (1977). Trimmed Spearman-Karber method for estimating median lethal concentrations in toxicity bioassays. Environ. Sci. Technol..

[83] Langford D.J., Bailey A.L., Chanda M.L., Clarke S.E., Drummond T.E., Echols S., Glick S., Ingrao J., Klassen-Ross T., Lacroix-Fralish M.L. (2010). Coding of facial expressions of pain in the laboratory mouse. Nat. Methods.

[84] Kiernan J.A., Dries D.J. (1999). Histological and Histochemical Methods: Theory and Practice.

